# Eye and Visual Health in New England: Findings from the Healthy Aging Data Reports

**DOI:** 10.1093/geroni/igab046.3181

**Published:** 2021-12-17

**Authors:** Shu Xu, Taylor Jansen, Chae Man Lee, Maki Karakida, Frank Porell, Elizabeth Dugan, Nina Silverstein

**Affiliations:** 1 University of Masschusetts Boston, Dorchester, Massachusetts, United States; 2 UMass Boston, Boston, Massachusetts, United States; 3 University of Massachusetts Boston, University of Massachusetts Boston, Massachusetts, United States; 4 University of Massachusetts Boston, Boston, Massachusetts, United States

## Abstract

Eye and visual health issues in older adults are prevalent, often undetected and untreated, but can contribute to poor physical and mental health issues, and higher mortality rates. The study describes state and local community rates of eye and visual health indicators (cataract, glaucoma, self-reported vision difficulty, and clinical diagnosis of blindness or visual impairment) of older adults 65+ in MA, NH, RI, and CT. Data sources used to calculate rates were: the American Community Survey (2014-2018 RI, 2012-2016 MA and NH, 2014-2018 CT) and the Medicare Current Beneficiary Summary File (2016-2017 RI, 2015 MA and NH, 2016-2017 CT). Small area estimation techniques were used to calculate age-sex adjusted community rates for more than 150 health indicators (https://healthyagingdatareports.org/). Disparities in rates were examined for 4 eye and visual health indicators: cataract, glaucoma, self-reported vision difficulty, and clinical diagnosis of blindness or visual impairment. Results showed variability in rates across states. MA had the highest rates of self-reported vision difficulty (5.8%) and blindness or visual impairment (1.5%), and the greatest differences in rates of self-reported vision difficulty (0.00-40.91%). CT had the highest rates of glaucoma (28.3%), and the greatest differences in rates of glaucoma (19.51-41.91%) and blindness or visual impairment (0.44-4.39%). RI had the highest rates of cataract (67.5%). Understanding the distribution of community rates makes disparities evident, and may help practitioners and policymakers to allocate resources to areas of highest need.

